# Transient Smartphone Blindness: Precaution Needed

**DOI:** 10.7759/cureus.1796

**Published:** 2017-10-24

**Authors:** Choudhary A Hasan, Fariha Hasan, Sayed Mustafa Mahmood Shah

**Affiliations:** 1 Department of Internal Medicine, Dow University of Health Sciences (DUHS), Karachi, Pakistan

**Keywords:** transient smartphone blindness

## Abstract

The transient smartphone blindness (TSB) is a relatively new phenomenon that affects vision. However, it warrants immediate attention by the physicians and the smartphone users, lest it leads to long-term consequences such as blindness.

## Editorial

In today’s world, the smartphones have become a necessity; Pakistan is no different. The ever-increasing number of the cell phone users in Pakistan has reached about 137 million, which includes a significant number of smartphone users [1].

A new phenomenon referred to as transient smartphone blindness (TSB) has emerged. This refers to temporary monocular vision loss associated with smartphone usage while lying down in the dark [2]. The symptoms reported by the patients are usually contralateral to the side on which they were lying. Blockage of light due to lying down caused one eye to undergo dark adaptation while the other (viewing) eye underwent light adaptation; this caused a temporary conflicting light adaptation between the two retinae [2]. When the blockage of light was removed, the light-adapted eye underwent monocular vision loss, lasting a couple of minutes in a patient and the reported time was 15 minutes [2]. This is a relatively unknown phenomenon with the potential for misdiagnosis as multiple sclerosis or ischemic vascular disease [3-4]. Following the diagnosis, the patients were started on therapy until the initial diagnosis was negated by further examinations [2-4]. In this case, the patients used their smartphones while in bed [2-4].

The current risk of developing the TSB is heightened by the ubiquitous use of the smartphones, lack of awareness of the effects of the smartphone by its users and the intensified blue light emitted by the latest smartphones. While the TSB itself is considered relatively benign and as a short-term pathology, the long-term implications arising from the disease have yet to be elucidated. Blue light emission from the smartphone has the potential to contribute to various retinal pathologies, such as age-related macular degeneration [5]. The disruptions in the sleep cycle by blue light exposure may also be exacerbated by smartphone usage in bed and thus promote the development of the TSB. The danger may arise from the initiation of inappropriate treatments following the misdiagnosis in the patients with ambiguous test results [3]. It is therefore crucial for the ophthalmologists and neurologists to include the history of the smartphone usage of the patient and the patients' daily practices before diagnosis. Future investigation into the TSB should assess disease frequency and the severity in those with predisposing conditions such as vitamin A deficiency or hereditary congenital stationary night blindness.

In order to prevent the TSB, people using smartphones should use them in well-lit rooms and in a position in which light does not enter only one eye. People should also try to avoid using their phones before going to bed in order to avoid disturbing their sleep cycles due to disruptive blue light emission. Another factor that could help overcome the alterations in sleep cycle from blue light is the use of technology that automatically reduces blue light for certain duration of time, this is currently used only by a few mobile phone manufacturers. Mobile phone manufacturers can spread awareness through public service messages warning their clientele of possible side effects of using phones in bed and in poorly lit rooms. The ophthalmologists can also create awareness about the TSB by counseling their patients especially teens. Further interventions may be provided in the form of mobile applications that monitor blue light exposure and offer prompts for prolonged or intense exposure.
